# Editorial: Impact evaluation using the translational science benefits model framework in the national center for advancing translational science clinical and translational science award program

**DOI:** 10.3389/fpubh.2025.1707595

**Published:** 2025-10-08

**Authors:** Pamela L. Davidson, Joe Hunt, Anna La Manna, Douglas A. Luke

**Affiliations:** ^1^CTSI-Evaluation, Clinical and Translational Science Institute (CTSI), University of California, Los Angeles, Los Angeles, CA, United States; ^2^Department of Health Policy and Management, Fielding School of Public Health, University of California, Los Angeles, Los Angeles, CA, United States; ^3^Indiana Clinical and Translational Sciences Institute (Indiana CTSI), Indiana University School of Medicine, Indianapolis, IN, United States; ^4^Center for Public Health Systems Science, School of Public Health, Washington University, St. Louis, MO, United States

**Keywords:** translational science, Translational Science Benefits Model (TSBM), knowledge translation, CTSA program, impact

## Introduction

Over the past several years, a new discipline has emerged called *translational science*. Translational science, championed by the National Center for Advancing Translational Science (NCATS), is defined as “…the field that generates innovations that overcome longstanding challenges along the translational research pipeline. These include scientific, operational, financial, and administrative innovations that transform the way that research is done, making it faster, more efficient, and more impactful” (1). In this sense, translational science is quite similar to the discipline of implementation science, which studies how evidence-based scientific knowledge is translated, adopted, implemented, and maintained in communities and healthcare settings (2).

Knowledge translation is a critical dissemination activity that transforms research results into new products, practices, and policies to benefit health and society. Yet, effectively bridging the gap between clinical research and practical applications is challenging. The US National Institutes of Health (NIH) and NCATS address this challenge through the Clinical and Translational Science Award (CTSA) Program. The charge of the CTSA Program is to transform the organization and infrastructure of the academic research enterprise to accelerate the movement of discoveries from clinical science to the bedside and community.

**Figure 1:**
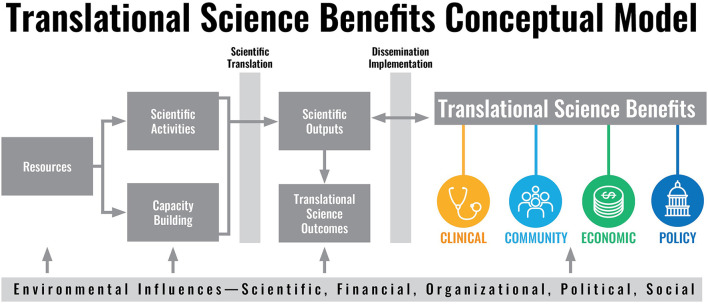
The Translational Science Benefits Model (TSBM) logic framework.

Documenting the results of these efforts is a necessary component to assess outcomes, health and social impacts, and support continuous improvement. Within the CTSA program, more than 60 CTSA hubs, primarily located at academic health sciences research institutes across the nation, are beginning to systematically measure and evaluate the impacts of their activities. One model used to track and assess impact is the Translational Science Benefits Model (TSBM), introduced in 2018, [see Figure 1, (3)]. TSBM is one of the pioneering frameworks for standardized documentation and dissemination of data on outcomes and impacts of translational science and translational research. Although translational science is still in its infancy, much work is being conducted nationally within CTSA hubs, communities, and research partners.

The recent *Frontiers in Public Health* Research Topic (*Impact evaluation using the translational science benefits model framework in the national center for advancing translational science clinical and translational science award program*) included 11 articles, written by more than 60 co-authors based at a wide range of CTSA hubs, contexts, and settings. Based on these articles, this editorial presents three crosscutting themes for reflecting on the TSBM Research Topic and state of the evolving science: 1) Versatility and methodological insights of the TSBM, 2) Knowledge translation as a pathway to longer-term impacts, and 3) Advancing translational science.

## Versatility and methodological insights of the TSBM

Articles in this edition demonstrate the versatility and methodological advancements of the TSBM. The TSBM began as a structured framework designed to assess the health and societal impacts of translational science. Over time, substantial advancements in methodology and operationalization have transformed TSBM into a dynamic tool for measuring the outcomes of translational science at multiple levels within the research enterprise, that also supports continuous improvement in scientific evaluation. This framework has been applied in various aspects such as setting strategic direction, operations management, continuous quality improvement (CQI), and tracking organizational contributions to advancing human health.

The integration of the TSBM framework has proven effective in eliciting recommendations for measures of significance on the contributions of the CTSA environment and consortium (Kane et al.). By integrating concept mapping with the TSBM framework, measures can be selected at both individual, organization and consortium levels, aiding strategic resource allocation.

For day-to-day management, the TSBM framework combined with the balanced scorecard and project management tools has supported organizational performance measurement at the program level, ensuring efficient allocation of resources and effective tracking of contributions to advancing priorities (Gholami et al.). Similarly, an adaptation of the TSBM framework in assessing performance has applied CQI tools to achieve performance improvements (Brimhall et al.). Methodological enhancements include real-time performance monitoring systems, balanced scorecards, and project management platforms, supporting frequent updates and comprehensive tracking aligned with strategic institutional goals (Swanson et al.).

Methodologies like Plan-Do-Study-Act cycles, inclusive leadership in team science, and automation through natural language processing (NLP) and artificial intelligence (AI) have further expanded the operational capabilities of the TSBM (Molzhon et al.). Concept mapping has aligned program goals with evaluation priorities (Manjunath et al.), ensuring diverse stakeholder perspectives are captured and consensus around evaluation metrics is formed (Kane et al.).

Overall, these advancements illustrate the evolution of the TSBM into a dynamic, methodologically robust measurement tool that effectively supports continuous improvement, providing standardized and comparative monitoring, and adaptive evaluation in translational science. This integration enables organizations to create and sustain a culture of impact, promoting awareness of the real-world benefits of their work (Davidson et al.).

## Knowledge translation: pathway to demonstrated health and societal impact

Potential and demonstrated knowledge translation impact is measured using four domains and 30 indicators operationalized in the TSBM. In a novel research project, CTSA hub-county interorganizational collaborations were followed longitudinally to assess both knowledge translation impact and the longer-term health and societal benefits reported in impact stories (Davidson et al.). Similarly, a Northern Ohio CTSA hub demonstrated societal benefits in public health practice, highlighting enhanced healthcare access, improving health outcomes, informing policy, and generating economic benefits (Zhang et al.). Researchers at the Duke University CTSA, shared the value of integrating TSBM into multiple levels of the research enterprise to examine impact, using case studies, program area level (e.g., pilot studies), and cross-program and institutional monitoring of TSBM in an organizational database (Sperling et al.). VCU CTSA evaluators emphasized increasing focus on educating investigators on the importance of measuring impact and the longer-term broad reaching effects of their translational science research (Molzhon et al.).

## Advancing translational science

As an impact evaluation framework, the TSBM is clearly relevant to translational science, given its goal of producing impactful scientific innovations. More specifically, a number of articles in this TSBM Research Topic feature one or more of the core principles of translational science (4). For example, the principle of *team science* is prominent in the article by Brimhall et al., who developed a logic model featuring inclusive leadership and other team science concepts. Methodological *creativity and innovation* were prominent in article which featured concept mapping (Kane et al.), innovative CQI methods including real-time monitoring dashboards (Gholami et al.), and natural language processing of bibliometric data (Manjunath et al.). Finally, a number of articles featured *boundary-crossing partnerships* between academic and community organizations in Los Angeles (Davidson et al.), Wisconsin and Missouri (Manjunath et al.), and a consortium of rural states (La Manna et al.).

## Conclusions

In summary, the CTSA Program, supported by NIH and NCATS, plays a pivotal role in transforming research into tangible health and societal benefits. The use of the TSBM has proven instrumental in systematically measuring and documenting these impacts. According to NCATS, “Translation turns observations in the laboratory, clinic, and community into diagnostics, therapeutics, medical procedures, and behavioral changes;” these are literally measured in the TSBM and are precursors to improvements in the delivery system and people's health.

Through advancements in methodology and versatile applications, the TSBM supports continuous improvement and adaptive evaluation, fostering a culture of impact within translational science. The Research Topic of articles in this edition reflects on the evolving state of translational science, underscoring its significance and the real-world benefits it provides.
